# Supplementary material for: COZOID: contact zone identifier for visual analysis of protein-protein interactions
Source: BMC Bioinformatics. 2018 Apr 6;19:125. doi: 10.1186/s12859-018-2113-6 (PMC5889581; doi:10.1186/s12859-018-2113-6)
Supplement: Supplementary file 2 — Software build. Executable binary file of the software tool. (ZIP 118,784 kb) [file 12859_2018_2113_MOESM2_ESM.zip › cozoid/platform/docs/testng-6.5.1-javadoc.zip!/org/testng/IConfigurationListener2.html]

IConfigurationListener2 (TestNG)


---


|  |  |  |
| --- | --- | --- |
| **Overview** \| **Package** \| **Class** \| **Tree** \| **Deprecated** \| **Index** \| **Help** | |  |
| **PREV CLASS**   **NEXT CLASS** | **FRAMES**    **NO FRAMES**     **All Classes** |  |
| SUMMARY: NESTED \| FIELD \| CONSTR \| METHOD | DETAIL: FIELD \| CONSTR \| METHOD |  |


---


## org.testng Interface IConfigurationListener2

**All Superinterfaces:**: IConfigurationListener, ITestNGListener

**All Known Implementing Classes:**: TestListenerAdapter, TestNG.ExitCodeListener

---

``` public interface IConfigurationListener2 extends IConfigurationListener ```

---

| **Method Summary** | |
| --- | --- |
| `void` | `beforeConfiguration(ITestResult tr)`             Invoked before a configuration method is invoked. |

**Methods inherited from interface org.testng.IConfigurationListener**

`onConfigurationFailure, onConfigurationSkip, onConfigurationSuccess`

**Method Detail**

### beforeConfiguration

```
void beforeConfiguration(ITestResult tr)
```

:   Invoked before a configuration method is invoked.


---


|  |  |  |
| --- | --- | --- |
| **Overview** \| **Package** \| **Class** \| **Tree** \| **Deprecated** \| **Index** \| **Help** | |  |
| **PREV CLASS**   **NEXT CLASS** | **FRAMES**    **NO FRAMES**     **All Classes** |  |
| SUMMARY: NESTED \| FIELD \| CONSTR \| METHOD | DETAIL: FIELD \| CONSTR \| METHOD |  |


---
